# Mononucleosis-like illnesses due to co-infection with severe fever with thrombocytopenia syndrome virus and spotted fever group rickettsia:a case report

**DOI:** 10.1186/s12879-021-06434-8

**Published:** 2021-08-18

**Authors:** Bin Zhao, Haohua Hou, Ran Gao, Bing Tian, Baocheng Deng

**Affiliations:** 1 Department of Infectious Diseases, The First Affiliated Hospital of China Medical University, No. 155 Nanjing North Street, Shenyang, 110001 Liaoning Province China; 2 Department of Infectious Diseases, Shengjing Hospital of China Medical University, Shenyang, 110000 Liaoning Province China; 3 Department of Hematology, The First Affiliated Hospital of China Medical University, Shenyang, 110001 Liaoning China

**Keywords:** Mononucleosis-like illnesses, Severe fever with thrombocytopenia syndrome virus, Spotted fever group rickettsia, Co-infection

## Abstract

**Background:**

We report a mononucleosis-like illnesses case due to co-infection with severe fever with thrombocytopenia syndrome virus (SFTSV) and spotted fever group rickettsia (SFGR), which to the best of our knowledge, has never been reported .

**Case presentation:**

A 64-year-old male with an 11-day history of fever, sore throat, malaise, nausea, and non-pruritic rash was admitted to our emergency department. Prior to admission, he was bitten by ticks. Laboratory tests revealed a white blood cell count of 24,460 cells/μL with 25% atypical lymphocytes and 20% mononucleosis, thrombocytopenia. Test results were positive for SFTSV RNA, SFTSV-specific IgM antibody, and SFGR-specific IgM antibody. He was diagnosed with mononucleosis-like illnesses due to co-infection with SFTSV and SFGR. After administration of doxycycline, he recovered completely.

**Conclusions:**

The clinical presentation may be atypical in co-infection with SFTSV and SFGR. This finding highlighted the importance of considering SFGR infection, as well as a SFSTV and SFGR co-infection for the differential diagnosis of patients bitten by ticks in SFTSV-endemic areas.

## Background

Severe fever with thrombocytopenia syndrome virus (SFTSV), a novel phlebovirus, renamed Huaiyangshan banyangvirus of the genus Banyangvirus, family *Phenuiviridae*, and order *Bunyavirales*, was first identified in China [1, 2]. Recently, SFTSV endemic areas have expanded from China to South Korea and western Japan, whereas the Heartland virus, which is closely related to SFTSV, was recently isolated in the US [3–5]. Thrombocytopenia (< 100 × 10^9^/L) and leucopenia (< 4.0 × 10^9^/L) appear to be consistent laboratory features of an SFTSV infection. Severe fever with thrombocytopenia syndrome (SFTS) caused by SFTSV infection is transmitted by ticks. Spotted fever group rickettsiae (SFGR) have been detected in *Haemaphysalis longicornis* ticks in China, which is also a vector of SFTSV [6, 7]. Occasionally, an SFGR and SFTSV co-infection can occur as a result of the same tick vector. Human infection with SFGR has not previously been reported in Liaoning Province, Northeast China; however, SFGR has been detected in hard ticks in Northeast China [8]. Infectious mononucleosis (IM) is a clinical syndrome caused by a primary Epstein-Barr virus (EBV) infection, which is common in young immune competent adults. IM is characterized by the classic triad of fever, pharyngitis, and lymphadenopathy. Laboratory tests are characterized by atypical lymphocytes and lymphocytosis. Moreover, EBV accounts for approximately 90% of clinical presentations suggestive of IM. Similar clinical presentations that are not attributed to EBV infections are referred to as “mononucleosis-like illnesses” (MLIs) [9]. Herein, we report the first case co-infected with SFTSV and SFGR in Northeast China, which presented with MLIs.

## Case presentation

A 64-year-old male with an 11-day history of fever, sore throat, malaise, nausea, and non-pruritic rash was admitted to our emergency department on June 4, 2013. Prior to admission, he was treated with cephalosporins and ibuprofen at a local hospital. On the sixth day of the illness, his condition worsened with the development of severe fever, leukocytosis, thrombocytopenia, and lymphadenopathy. He lived in an urban area and was previously healthy. The patient reported that he had visited a hilly region of Jilin Province adjacent to Eastern Liaoning Province 2 weeks prior, where he was bitten by a tick.

**Fig. 1:**
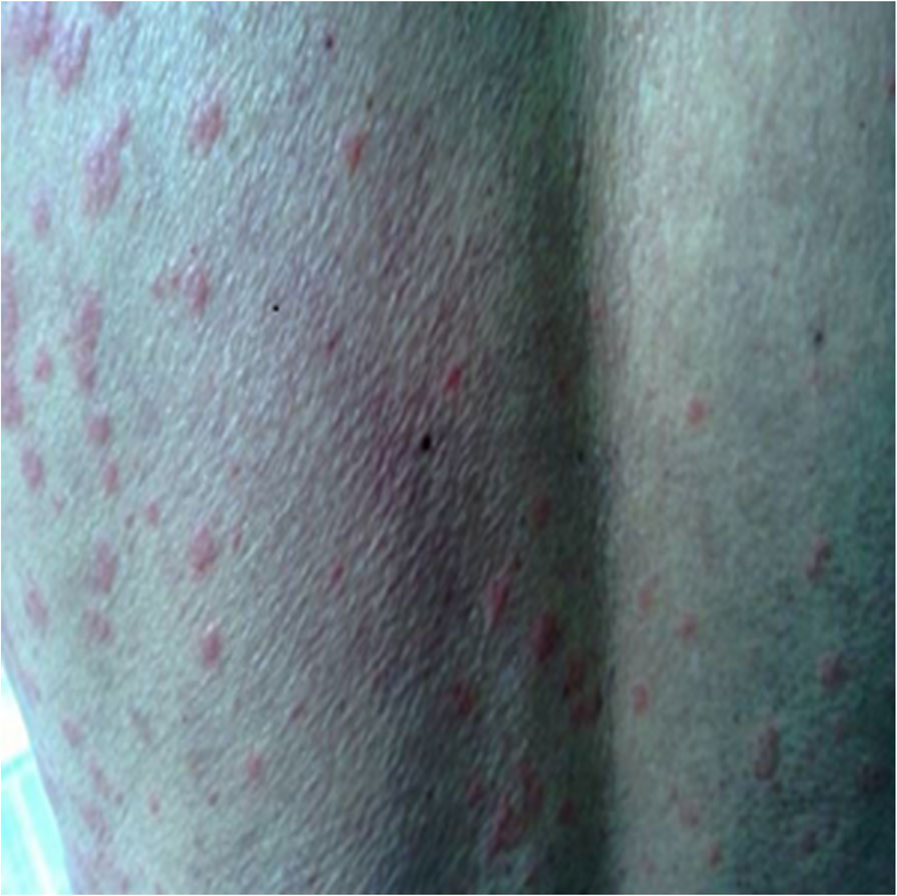
Non-pruritic rash on the patient’s back

Upon admission, his general status was moderate; a physical examination revealed a high fever (axillary temperature of 39.5 °C), throat congestion, exudative tonsillitis, and lymphadenopathy with maculopapular rashes and no eschar. The initial laboratory studies revealed a white blood cell count of 24,460 cells/μL with 25% atypical lymphocytes and 20% mononucleosis, thrombocytopenia, and increased immunoglobulin (Ig) G (Table 1). Ultrasonography and computed tomography revealed severe lymphadenopathy in the neck, groin, abdomen, and mediastinum. Serum samples were collected on day 12 of the illness to perform a serological differential diagnosis (Fig. 1).

**Table 1:** Laboratory test results on admission

Laboratory test values on admission	Value	Reference values
**CBC**		
Leukocytes (n/mm^3^)	24,460	3500–9, 500
Neutrophils (n/mm^3^)	9050 (37%)	1800–6, 300
Lymphocytes (n/mm^3^)	2446 (10%)	1100–3, 200
Monocytes (n/mm^3^)	4892 (20%)	100–600
atypical lymphocytes (n/mm^3^)	6115 (25%)	0
Hemoglobin (g/dL)	12.3	13.0–17.5
Platelets (n/mm^3^)	75,000	125,000-350,000
CRP (mg/L)	75.90	0.00–8.00
**Biochemical tests**		
Alanine aminotransferase (U/L)	37	13–69
Aspartate aminotransferase (U/L)	28	15–46
Lactate dehydrogenase (U/L)	794	313–618
Alkaline phosphatase (U/L)	244	38–126
γ-GGT (U/L)	75	12–58
Albumin (g/L)	31.0	35.0–50.0
Total protein (g/L)	79.2	63.0–82.0
Na^+^ (mmol/L)	134.4	136–145
**Coagulation**		
Activated partial thromboplastin time (s)	68.3	31.5–43.5
International normalized ratio	1.42	0.82–1.15
**Urinalysis**		
Protein	+	Negative
pH	5.5	5.0–7.0
WBC (n/optical field)	2.40	0.00–2.08
RBC (n/optical field)	1.29	0.00–1.98

Paired sera on the 11th and 18th day of onset were test for cytomegalovirus (CMV), human herpesvirus 6 (HHV-6), herpes simplex virus-1 and 2, *Brucella*, and the protozoan *Toxoplasma gondii* were positive only for IgG antibodies to CMV. Antibodies specific for Hantaan virus, Crimean-Congo hemorrhagic fever virus (CCHFV), and various respiratory viruses (adenovirus, respiratory syncytial viruses A and B, influenza viruses A and B, and parainfluenza virus types 1, 2, and 3) in paired sera were negative. The samples were positive for Epstein-Barr viral capsid IgG and EBV nuclear antigen IgG antibodies. The results of the heterophile agglutination test were negative. The polymerase chain reaction (PCR) analyses were negative for typhus, scrub typhus, EBV, CMV, HHV-6, human granulocytic anaplasmosis, *Leptospir*a, and CCHFV. Human immunodeficiency virus- and hepatitis virus-specific antibodies were negative at the time of admission. The test results for antinuclear antibodies, circulating immune complexes and anti-neutrophil cytoplasmic antibodies were also negative. The stool, blood, and urine samples were negative for routine bacterial cultures; however, the throat cultures were negative. A bone marrow biopsy performed on day 13 of disease onset was unremarkable.

The patient was diagnosed with MLIs; however, the specific pathogen remained unidentified. Liaoning Province is the only SFTSV-endemic area in Northeast China [10]. The patient visited a hilly area adjacent to east Liaoning Province prior to symptom onset and reported being bitten by a tick. Although the patient’s presentation was not typical SFTS, the serum samples collected on day 13 of the illness were sent to the diagnostic laboratory of infectious diseases of the Centers of Disease Control in Liaoning Province for SFTSV analysis. These samples were positive for SFTSV by real-time PCR, with RNA loads of 2.5 × 10^6^ copies/mL. The test result was positive for SFTSV-specific IgM antibody, but negative for SFTSV-specific IgG antibody. Thus, the patient was diagnosed with MLIs attributed to SFTSV infection.

The patient received nutritional support therapy and fluid supplementation upon admission. On hospitalization day 3, intravenous Ig was initiated at a dose of 30 g/day for 5 days. The patient’s condition did not improve. SFTS is characterized by fever, thrombocytopenia, leukocytopenia, and lymphadenopathy; however, the patient presented with MLIs, which was not consistent with the typical clinical manifestations of an SFTSV infection. Since ticks can carry multiple pathogens, including emerging tick-associated agents in mainland China [11], SFTSV co-infection with other tick-borne agents (e.g., SFGR and *B burgdorferi sensulato*) was under consideration. However, the test for SFGR was not available in our laboratory at the time. Since doxycycline is the recommended therapeutic regimen for rickettsia, anaplasma, ehrlichia, and *Borrelia burgdorferi sensulato* infections, we prescribed doxycycline at a dosage of 0.1 mg (twice daily) to cover other tick-borne pathogens. The body temperature returned to normal within 12 h following the administration of doxycycline, and continued for 2 weeks after the body temperature returned to normal. The leukocyte count returned to normal levels, and the patient was discharged on day 7 post-admission. At the 6-month follow-up, recovery was complete.

In 2016, co-infection with SFTS and SFGR was reported in SFTS endemic area in Central China [12]. In 2019, we retrospectively investigated SFGR infection among SFTS patients whose blood samples on admission were stored at − 80ºC since 2010. Real-time PCR and an ELISA (Spotted fever rickettsia IgM Antibody Kit, FULLER) were performed. The patient’s serum collected on the eleventh day of onset 6 years prior, was positive for SFGR-specific IgM antibody and negative for SFGR-specific IgG antibody. The PCR test for *B burgdorferi sensulato* was negative. The patient was finally retrospectively diagnosed with SFTSV co-infection with SFGR 6 years after disease onset.

## Discussion and conclusions

Concurrent infection with two emerging tick-borne pathogens, SFTSV and SFGR, was documented by serological and molecular methods in this patient. SFTSV co-infection with other tick-borne pathogens may occur from the same tick bite. This patient is the first SFGR-infected patient in Liaoning Province, Northeast China, who was simultaneously co-infected with SFTSV. SFTS patients typically present with fever, gastrointestinal symptoms, hemorrhagic signs, or influenza-like illness upon the acute onset of disease, which can range from mild and self-limiting disease to severe disease with life-threatening complications [10]. Because rickettsial infections are clinically similar to several viral infections, they are difficult to distinguish from a viral infection [13]. A rash is more common in SFGR than SFTS, whereas thrombocytopenia (< 100 × 10^9^/L) and leucopenia (< 4.0 × 10^9^/L) appear to be consistent features of an SFTSV infection [14]. The study by Lu et al. reported that 82.2 and 83.7% of patients co-infected with SFTSV and SFGR presented with leucopenia and thrombocytopenia, respectively [12]. This case also differed from other SFTSV and rickettsia co-infected cases, which have never been reported as MLIs.

A diagnosis of IM is frequently based on clinical presentation, the presence of atypical lymphocytes on a peripheral blood smear, and positive heterophile antibody test. Serological testing for the identification of antibodies against EBV-specific antigens is required to establish a definite diagnosis. Real-time PCR is a useful tool to arrive at an early diagnosis of IM in cases involving inconclusive serological results [15]. However, 10% of IM cases cannot be attributed to an EBV infection. The only positive Epstein-Barr viral capsid IgG and EBV nuclear antigen IgG antibodies, and negative results of heterophile agglutination test and EBV DNA indicated past EBV infection for this case. Bacterial, protozoal, and herpesvirus infections are referred to as MLIs, which are a rare manifestation. In addition, systemic disorders (e.g., sarcoidosis and malignancies) can also cause MLIs [9]. The symptoms of the present case were clinically similar to those of IM, in laboratory and clinical presentations (e.g., lymphadenopathy, skin rash, pharyngitis, and splenomegaly), which differ from those of a typical SFTSV infection. The patient could have been misdiagnosed if the epidemiological data had been ignored (i.e., history of tick bite and location of the bite). Although SFTSV infection was confirmed in this patient, co-infection with other tick-borne pathogens was also under consideration due to the atypical clinical presentation. Because doxycycline is the recommended therapeutic regimen for rickettsia, anaplasma, ehrlichia, and *Borrelia burgdorferi sensulato* infection, we prescribed doxycycline to cover other tick-borne pathogens. At 12 h post-doxycycline treatment, the patient’s body temperature quickly returned to normal, and his condition quickly improved. A retrospective SFGR infection test 6 years after admission proved that the patient suffered from a co-infection with SFTS and SFGR, and the treatment regimen was appropriate.

The symptoms common to SFTSV- and SFGR-infected patients are not intensified in patients with an SFTSV and SFGR co-infection in Henan Province, which is a SFTS-endemic area located in Central China [12]. Less common hemorrhagic signs, especially gastrointestinal hemorrhages, are exacerbated in SFTSV and SFGR co-infected patients than in SFSV single-infected patients [12]. Despite a prolonged international normalized ratio, the patient did not exhibit any hemorrhagic signs, which was partially attributable to a decreased moderate platelet count in this patient. Nonetheless, the pathogenesis of SFTS remains poorly understood. A cytokine storm is associated with increased disease severity [16, 17]. We suspected that the thrombocytopenia observed in this patient was due to phagocytosis by splenic macrophages [18]. Leukocytosis may be observed when a secondary bacterial infection occurs in conjunction with SFTS. Moreover, an SFTSV infection associated with aplastic anemia and hemophagocytic lymphohistiocytosis has also been reported [10].

The current recommended therapeutic regimen for rickettsiosis is the administration of tetracyclines, and doxycycline is the preferred agent due to its dosage (twice daily) and superior patient tolerance [19]. To date, no specific antiviral agents are available for the treatment of SFTSV. The early diagnosis and treatment of SFTSV infection can the improve clinical outcomes. Although ribavirin can inhibit SFTSV activity in vitro, it has little clinical efficacy for the treatment of SFTSV infection [10, 20, 21]. Ig therapy may be efficacious against SFTSV infection [22]. In addition, plasma exchange is a potential therapy for severe SFTSV infection [23].

To the best of our knowledge, this case is the first report of MLI induced by co-infection with SFTSV and SFGR. This is also the first case of an SFGR-infected patient in Liaoning Province, Northeast China. Compared with SFTSV or SFGR infection, the clinical presentation may be atypical in co-infection with SFTSV and SFGR. This finding highlighted the importance of considering SFGR infection, as well as a SFSTV and SFGR co-infection for the differential diagnosis of patients bitten by ticks in SFTSV-endemic areas.

## Data Availability

All the information supporting our conclusions are included in the manuscript. There are no datasets related to this case report.

## Abbreviations

IM: Infectious mononucleosis
EBV: Epstein-barr virus
MLIs: Mononucleosis-like illnesses
SFTSV: Severe fever with thrombocytopenia syndrome virus
SFTS: Severe fever with thrombocytopenia syndrome
SFGR: Spotted fever group rickettsiae
IgG: Immunoglobulin G
CMV: Cytomegalovirus
HHV-6: Human herpesvirus 6
PCR: Polymerase chain reaction

## References

[1] Yu XJ, Liang MF, Zhang SY, Liu Y, Li JD, Sun YL, Zhang L, Zhang QF, Popov VL, Li C, Qu J, Li Q, Zhang YP, Hai R, Wu W, Wang Q, Zhan FX, Wang XJ, Kan B, Wang SW, Wan KL, Jing HQ, Lu JX, Yin WW, Zhou H, Guan XH, Liu JF, Bi ZQ, Liu GH, Ren J, Wang H, Zhao Z, Song JD, He JR, Wan T, Zhang JS, Fu XP, Sun LN, Dong XP, Feng ZJ, Yang WZ, Hong T, Zhang Y, Walker DH, Wang Y, Li DX (2011). Fever with thrombocytopenia associated with a novel bunyavirus in China. N Engl J Med.

[2] Kuhn JH, Adkins S, Alioto D, Alkhovsky SV, Amarasinghe GK, Anthony SJ, Avsic-Zupanc T, Ayllon MA, Bahl J, Balkema-Buschmann A (2020). 2020 taxonomic update for phylum Negarnaviricota (Riboviria: Orthornavirae), including the large orders Bunyavirales and Mononegavirales. Arch Virol.

[3] Xing Z, Schefers J, Schwabenlander M, Jiao Y, Liang M, Qi X, Li C, Goyal S, Cardona CJ, Wu X, Zhang Z, Li D, Collins J, Murtaugh MP (2013). Novel bunyavirus in domestic and captive farmed animals, Minnesota, USA. Emerg Infect Dis.

[4] Shimojima M, Fukushi S, Tani H, Yoshikawa T, Morikawa S, Saijo M (2013). Severe fever with thrombocytopenia syndrome in Japan. Uirusu.

[5] Kim KH, Yi J, Kim G, Choi SJ, Jun KI, Kim NH, Choe PG, Kim NJ, Lee JK, Oh MD (2013). Severe fever with thrombocytopenia syndrome, South Korea, 2012. Emerg Infect Dis.

[6] Liu W, Li H, Lu QB, Cui N, Yang ZD, Hu JG, Fan YD, Guo CT, Li XK, Wang YW (2016). Candidatus Rickettsia tarasevichiae Infection in Eastern Central China a case series. Ann Intern Med.

[7] Shao JW, Zhang XL, Li WJ, Huang HL, Yan J. Distribution and molecular characterization of rickettsiae in ticks in Harbin area of Northeastern China. Plos Neglect Trop D. 2020;14(6):e0008342. 10.1371/journal.pntd.0008342 PMC7272007 32497120

[8] Guo WP, Wang YH, Lu Q, Xu G, Luo Y, Ni X, Zhou EM (2019). Molecular detection of spotted fever group rickettsiae in hard ticks, northern China. Transbound Emerg Dis.

[9] Hurt C, Tammaro D (2007). Diagnostic evaluation of mononucleosis-like illnesses. Am J Med.

[10] Deng B, Zhou B, Zhang S, Zhu Y, Han L, Geng Y, Jin Z, Liu H, Wang D, Zhao Y, Wen Y, Cui W, Zhou Y, Gu Q, Sun C, Lu X, Wang W, Wang Y, Li C, Wang Y, Yao W, Liu P (2013). Clinical features and factors associated with severity and fatality among patients with severe fever with thrombocytopenia syndrome Bunyavirus infection in Northeast China. PLoS One.

[11] Fang LQ, Liu K, Li XL, Liang S, Yang Y, Yao HW, Sun RX, Sun Y, Chen WJ, Zuo SQ, Ma MJ, Li H, Jiang JF, Liu W, Yang XF, Gray GC, Krause PJ, Cao WC (2015). Emerging tick-borne infections in mainland China: an increasing public health threat. Lancet Infect Dis.

[12] Lu QB, Li H, Zhang PH, Cui N, Yang ZD, Fan YD, Cui XM, Hu JG, Guo CT, Zhang XA, Liu W, Cao WC (2016). Severe fever with thrombocytopenia syndrome complicated by co-infection with spotted fever group Rickettsiae, China. Emerg Infect Dis.

[13] Walker DH, Paddock CD, Dumler JS (2008). Emerging and re-emerging tick-transmitted rickettsial and ehrlichial infections. Med Clin N Am.

[14] Liu Q, He B, Huang SY, Wei F, Zhu XQ (2014). Severe fever with thrombocytopenia syndrome, an emerging tick-borne zoonosis. Lancet Infect Dis.

[15] Vouloumanou EK, Rafailidis PI, Falagas ME (2012). Current diagnosis and management of infectious mononucleosis. Curr Opin Hematol.

[16] Deng B, Zhang S, Geng Y, Zhang Y, Wang Y, Yao W, Wen Y, Cui W, Zhou Y, Gu Q, Wang W, Wang Y, Shao Z, Wang Y, Li C, Wang D, Zhao Y, Liu P (2012). Cytokine and chemokine levels in patients with severe fever with thrombocytopenia syndrome virus. PLoS One.

[17] Sun Y, Jin C, Zhan F, Wang X, Liang M, Zhang Q, Ding S, Guan X, Huo X, Li C, Qu J, Wang Q, Zhang S, Zhang Y, Wang S, Xu A, Bi Z, Li D (2012). Host cytokine storm is associated with disease severity of severe fever with thrombocytopenia syndrome. J Infect Dis.

[18] Jin C, Liang M, Ning J, Gu W, Jiang H, Wu W, Zhang F, Li C, Zhang Q, Zhu H, Chen T, Han Y, Zhang W, Zhang S, Wang Q, Sun L, Liu Q, Li J, Wang T, Wei Q, Wang S, Deng Y, Qin C, Li D (2012). Pathogenesis of emerging severe fever with thrombocytopenia syndrome virus in C57/BL6 mouse model. Proc Natl Acad Sci U S A.

[19] Jia N, Zheng YC, Ma L, Huo QB, Ni XB, Jiang BG, Chu YL, Jiang RR, Jiang JF, Cao WC (2014). Human infections with rickettsia raoultii, China. Emerg Infect Dis.

[20] Liu W, Lu QB, Cui N, Li H, Wang LY, Liu K, Yang ZD, Wang BJ, Wang HY, Zhang YY, Zhuang L, Hu CY, Yuan C, Fan XJ, Wang Z, Zhang L, Zhang XA, Walker DH, Cao WC (2013). Case-fatality ratio and effectiveness of ribavirin therapy among hospitalized patients in China who had severe fever with thrombocytopenia syndrome. Clin Infect Dis.

[21] Shimojima M, Fukushi S, Tani H, Yoshikawa T, Fukuma A, Taniguchi S, Suda Y, Maeda K, Takahashi T, Morikawa S, Saijo M (2014). Effects of ribavirin on severe fever with thrombocytopenia syndrome virus in vitro. Jpn J Infect Dis.

[22] Ye J (2012). Severe fever with thrombocytopenia syndrome expert consensus. Chin J Exp Clin Infect Dis.

[23] Oh WS, Heo ST, Kim SH, Choi WJ, Han MG, Kim JY (2014). Plasma exchange and ribavirin for rapidly progressive severe fever with thrombocytopenia syndrome. Int J Infect Dis.

